# Pain Sensation and Postsurgical Complications in Posterior Mandibular Implant Placement Using Ridge Mapping, Panoramic Radiography, and Infiltration Anesthesia

**DOI:** 10.1155/2013/134210

**Published:** 2013-05-21

**Authors:** Ali Saad Thafeed AlGhamdi

**Affiliations:** Faculty of Dentistry, King Abdulaziz University, P.O. Box 109725, Jeddah 21351, Saudi Arabia

## Abstract

*Objectives*. The aim of this study was to investigate intrasurgical and after surgical, pain and the incidence of after surgical alteration of sensation in the mandible and lower lip when placing implants in the posterior mandible using ridge mapping, panoramic radiography, and infiltration anesthesia. *Methods*. This was a longitudinal clinical study of healthy patients needing implant placement in the posterior mandible. After thorough examination and treatment plan using ridge mapping and panoramic radiography, all patients received dental implants under local infiltration anesthesia. The patients were then given a questionnaire to assess the pain during anesthesia and implant surgery. Change of sensation in the lower lip was evaluated by standard neurosensory examination tests at 7 days and 1 and 4 months. Prosthetic treatment was carried out 4 months postsurgery and the patients were followed for an average of 28.5 months afterwards. *Results*. A total of 103 implants were placed in 62 patients. Patients reported very minor pain during injection. No pain was reported during either implant placement or bone grafting procedures. No alteration of sensation in the mandible or lower lip was recorded postsurgery. *Conclusion*. In most cases, ridge mapping, panoramic radiography, and infiltration anesthesia are sufficient for posterior mandibular implant placement without pain or complications.

## 1. Introduction

Dental implantology has become a widely accepted method of treatment. Because of its ability to accurately restore aesthetics and function, it has become the preferred option for replacing missing teeth. The long-term clinical success of the implant depends on accurate presurgical planning, careful surgical technique, and proper prosthetic design. The goal of presurgical planning is to accurately position the implant while keeping in mind the location of vital anatomical structures such as the inferior alveolar nerve, mental foramen, and maxillary sinus [1, 2]. 

Despite the high success rate of dental implants, many complications have been encountered with their placement. One of the most serious complications is the alteration of sensation after placement in the posterior mandible. The prevalence of such a complication has been reported to be as high as 13% [3, 4]. This can occur as a result of injury to the inferior alveolar nerve (IAN) from traumatic local anesthetic injections or, most importantly, during dental implant osteotomy or placement [1, 5]. This complication is one of the most unpleasant patient experiences, so every precaution should be taken to avoid it [1]. 

Traumatic local anesthetic injection can cause injury to the nerve directly through the needle or as a result of hematoma formation or neurotoxicity from the anesthetic solution [6, 7]. To avoid this, local infiltration anesthesia during implant placement in the mandibular posterior area has been recommended [7–9].

Several methods are used to localize the IAN during treatment planning. These include panoramic radiography, computed tomography, and cone beam computed tomography (CBCT) [10]. CBCT provides the most accurate method for localizing the IAN. Its high cost and level of radiation prevent it from becoming the standard of care. Most clinicians use conventional radiography (e.g., panoramic, periapical) to localize the IAN, which is sufficient in many cases [11–14]. Panoramic radiography can be used safely for many cases but with some limitations. A 2 mm safety zone between the apical part of the implant and the upper border of the IAN canal is highly recommended by most implant practitioners [3, 15]. The magnification of the X-ray machine must be known; some recommend placing an object of known dimension in the mouth before taking the radiograph. This technique allows accurate calculation of the dimensional changes in the panoramic radiograph [1]. 

Because conventional radiography produces only a 2-dimensional record, other methods must be used to overcome this problem. Ridge mapping and bone sounding under local anesthesia are helpful in determining the buccolingual width of the ridge. In some cases, the crest of the ridge is too thin, and the implant surgeon should consider these few millimeters during dental implant planning useless if used for implant support [16].

The aim of the current study was to investigate intrasurgical and postsurgical pain as well as the incidence of postsurgical alteration of sensation in the mandible and lower lip when placing an implant in the posterior mandible using ridge mapping, panoramic radiography, and infiltration anesthesia. 

## 2. Materials and Methods

### 2.1. Study Design

This was a longitudinal clinical study. The study design was reviewed and approved by the Ethics Committee of the Faculty of Dentistry, King Abdulaziz University, Jeddah, Saudi Arabia. 

### 2.2. Study Population

Healthy patients who needed dental implants placed in the posterior mandibular area participated in the study. Patients were advised of their role in the study, possible postsurgical complications, and advantages and disadvantages of the surgical procedures. They signed an informed consent to participate in the study.

### 2.3. Exclusion Criteria

The exclusion criteria include the following:

1. patients needing extraction and immediate implant placement,
2. smokers,
3. patients with medical problems such as diabetes, osteoporosis, blood dyscrasias, and malignancies,
4. patients with poor oral hygiene,
5. patients with altered sensation in the lower lip due to previous mandibular surgery or third molar extraction [17],
6. patients with untreated periodontal disease,
7. patients with severe class I ridge defects and class II or III ridge defects in the surgical area [18].

### 2.4. Study Protocol

Data including patient age, sex, medical history, dental history, oral hygiene practice, distance from crest of the ridge to the inferior alveolar nerve, buccolingual and apicocoronal dimensions of the bone in the surgical area, type of bone graft needed (if any), length of implant used, healing process, postsurgical complications, time before loading, postimplant bone loss, type of prosthesis used, and time of followup were recorded. 

The buccolingual dimension of the bone in the surgical area was measured by ridge mapping using the Wilson bone caliper [19]. Based on the remaining bone thickness, the decision was made about the need for bone grafting. 

Diagnostic records (panoramic radiograph, periapical radiograph, and diagnostic casts) were taken before surgery. From the panoramic radiograph, the amount of available bone (clinical bone height) was calculated using the formula proposed by Alhassani and AlGhamdi [1]. The 2 mm safety zone between the apical part of the implant and the superior border of the IAN was subtracted from the clinical bone height. Because the implant drill is slightly longer than the implant, an additional 0.5 mm was subtracted. If the crest of the ridge contained very thin bone that could not be used to support the implant, this was subtracted, too, and the implant length was determined accordingly. 

Patients were given a questionnaire to evaluate pain during anesthesia and implant surgery. The severity of pain was assessed by using a 10 cm visual analogue scale, labeled as “no pain” at the zero extreme and “severe pain” at the 10 cm mark. Changes of sensation in the lower lip were evaluated by standard neurosensory examination tests [1] at 7 days and 1 and 4 months. 

### 2.5. Surgical Procedures

The patients were given 1 g of amoxicillin 1 hour before surgery and 500 mg every 8 hours for 1 week. 

Treatment was carried out under local anesthesia with local infiltration buccally and lingually. A crestal incision was made in the surgical area and divergent releasing incisions remote to the defect area were used if needed. A full thickness flap was elevated. The proposed implant site was prepared according to the manufacturer's recommendation, and the implant with the desired length was placed in an ideal position about 3 mm apical to the cementoenamel junction of the adjacent teeth. Guided bone regeneration was used if needed (mixture of bovine bone (particle size, 0.25–1.0 mm) and CaSO_4_ (ratio, 4 : 1), covered with a layer of CaSO_4_) [20, 21]. A healing abutment was placed. The flap was secured using 4-0 vicryl interrupted sutures. Patients were given NSAIDS (ibuprofen, 600 mg) and chlorhexidine mouth wash for 1 week after surgery. 

### 2.6. Evaluation of Healing

Sutures were removed after 7 days. Soft-tissue healing was monitored carefully during the healing period to evaluate any early or late complications at the surgical site and the effect of these complications, if any, on implant success. The patients were reevaluated after 1 and 4 months. 

### 2.7. Prosthetic Treatment

Patients were sent to the Prosthodontic Department for final restoration 4 months after implant placement and were followed for an average of 28.5 months afterward (range, 12–60 months). 

## 3. Results

A total of 103 implants were placed for 62 patients (22 males and 40 females). The mean age of the study group was 42.85 ± 13.74 years. The distribution of implants and their lengths are shown in Table 1. Of the implants placed, 7.8% were 10 mm long, 23.3% were 11.5 mm, and 68.9% were 13 mm. About 60.2% of the implants were placed in the first molar region, 18.4% in the second premolar region, 17.5% in the second molar region, and 3.9% in the first premolar region. According to the presurgical ridge mapping, we found that horizontal bone loss was mild in 10 sites and moderate in 33. Similar findings were reported during surgery. Cases with mild horizontal ridge defects did not require bone grafting because the remaining bone was sufficient for implant placement, while cases with moderate horizontal bone loss required bone grafting after implant placement. The surgical procedures were performed without difficulty. Good primary stability was obtained for all implants. Patients reported only minimal pain during injection. No pain was reported during implant placement or bone grafting procedures.

Patients reported only minor discomfort during the second day after surgery; this was managed by analgesics, with no pain or discomfort reported afterward. No alteration of sensation in the mandible or lower lip was recorded after surgery. The surgical sites healed without complication or infection following implant placement. All cases showed excellent clinical stability.

Single crowns were placed on 65 implants, 31 became abutments for fixed partial dentures, and 7 were abutments for hybrid prostheses. At 12 months after loading, crestal bone loss ranged from 0.4 to 1.2 mm (mean, 0.77 ± 0.34 mm) as demonstrated by periapical radiographs, and pocket depth ranged from 2 to 4 mm (mean, 2.6 ± 0.56 mm). No significant difference in crestal bone loss and pocket depth was noticed between males and females and between patients with and without bone grafting. No significant change in crestal bone loss or pocket depth was noticed afterwards. 

## 4. Discussion

Presurgical planning is of paramount importance for successful dental implant treatment. Combining clinical examination and radiographic analysis is essential for proper presurgical evaluation of implant sites.

Ridge mapping allows accurate measurement of alveolar bone thickness in the edentulous area prior to implant placement. It is a simple and predictable procedure, providing a measurement of bone thickness consistent with those obtained following surgical exposure of bone [19, 22–24]. Chen et al. [22] reported that CBCT was less consistent than ridge mapping in measuring the buccolingual thickness of bone in the edentulous area. In the current study, ridge mapping was very consistent with direct measurements. Presurgical planning based on ridge mapping was similar to what was done during surgery.

Clinicians who depend mainly on the panoramic radiograph for localizing the IAN must keep in mind the inherent magnification of this instrument. In the current study, we followed the protocol of Alhassani and AlGhamdi [1] to calculate the magnification factor and clinical bone height. We subtracted 2 mm from the clinical bone height as a safety zone, then 0.5 mm because the drill is slightly longer than the implant, and then the implant length was estimated. No change in implant length was made during surgery from what was planned presurgically. The space between the apex of the implant and the superior border of the IAN was similar to the presurgical estimate, and there was no nerve injury or change in sensation reported postsurgically. This indicates that panoramic radiography is a safe and predictable method for presurgical planning of dental implant placement in the posterior mandible, provided that the magnification factor and the 2 mm safety zone between the apical part of the osteotomy and the upper boarder of the IAN are kept in mind. It is also important to position the patient in the panoramic machine correctly and combine the clinical and radiographic findings [2, 11–14].

Heller and Shankland II (2001) [8] advocated using local infiltration for anesthesia instead of an IAN block while placing implants in the posterior mandible. They proposed that without complete lack of sensation, the patient will feel pain if the drill approaches the IAN. Using infiltration anesthesia will also eliminate the risk of IAN injury from traumatic local anesthetic injection. However, this technique is not used widely because the bone has sensitive nerve endings that may cause discomfort during surgery [7, 8]. In the current study, all patients received infiltration anesthesia. None of the patients reported any pain during surgery, indicating that local infiltration is safe and sufficient for implant placement in the posterior mandible. 

All implants were successful, having no nerve injury or change in sensation of the mandible or lower lip. Various prosthetic designs were used for these implants without any long-term complications.

## 5. Conclusion

In most cases, ridge mapping, panoramic radiography, and infiltration anesthesia are sufficient for placement of dental implants in the posterior mandible without pain or postsurgical complications. 

## Figures and Tables

**Table 1:** Length and distribution of implants.

Tooth number	34	35	36	37	44	45	46	47	Total
Implant length, mm									
10	0	4	1	2	0	1	0	0	8
11.5	0	4	7	3	0	2	5	3	24
13	1	5	19	3	3	3	30	7	71
15	0	0	0	0	0	0	0	0	0

Total number of implants	1	13	27	8	3	6	35	10	103

## References

[1] Alhassani AA, AlGhamdi AS (2010). Inferior alveolar nerve injury in implant dentistry: diagnosis, causes, prevention, and management. *The Journal of Oral Implantology*.

[2] Mehra A, Pai KM (2009). Evaluation of dimensional accuracy of panoramic cross-sectional tomography, its ability to identify the inferior alveolar canal, and its impact on estimation of appropriate implant dimensions in the mandibular posterior region. *Clinical Implant Dentistry and Related Research*.

[3] Bartling R, Freeman K, Kraut RA (1999). The incidence of altered sensation of the mental nerve after mandibular implant placement. *Journal of Oral and Maxillofacial Surgery*.

[4] Ellies LG (1992). Altered sensation following mandibular implant surgery: a retrospective study. *The Journal of Prosthetic Dentistry*.

[5] Hegedus F, Diecidue RJ (2006). Trigeminal nerve injuries after mandibular implant placement—practical knowledge for clinicians. *International Journal of Oral and Maxillofacial Implants*.

[6] Moon S, Lee SJ, Kim E, Lee CY (2012). Hypoesthesia after IAN block anesthesia with lidocaine: management of mild to moderate nerve injury. *Restorative Dentistry & Endodontics*.

[7] Juodzbalys G, Wang HL, Sabalys G, Sidlauskas A, Galindo-Moreno P (2011). Inferior alveolar nerve injury associated with implant surgery. *Clinical Oral Implants Research*.

[8] Heller AA, Shankland WE (2001). Alternative to the inferior alveolar nerve block anesthesia when placing mandibular dental implants posterior to the mental foramen. *The Journal of Oral Implantology*.

[9] Levitt DS (2003). Apicoectomy of an endosseous implant to relieve paresthesia: a case report. *Implant Dentistry*.

[10] Anderson LC, Kosinski TF, Mentag PJ (1991). A review of the intraosseous course of the nerves of the mandible. *The Journal of Oral Implantology*.

[11] Frei C, Buser D, Dula K (2004). Study on the necessity for cross-section imaging of the posterior mandible for treatment planning of standard cases in implant dentistry. *Clinical Oral Implants Research*.

[12] Garg AK, Vicari A (1995). Radiographic modalities for diagnosis and treatment planning in implant dentistry. *The Implant Society*.

[13] Tal H, Moses O (1991). A comparison of panoramic radiography with computed tomography in the planning of implant surgery. *Dentomaxillofacial Radiology*.

[14] Vazquez L, Saulacic N, Belser U, Bernard JP (2008). Efficacy of panoramic radiographs in the preoperative planning of posterior mandibular implants: a prospective clinical study of 1527 consecutively treated patients. *Clinical Oral Implants Research*.

[15] Kraut RA, Chahal O (2002). Management of patients with trigeminal nerve injuries after mandibular implant placement. *Journal of the American Dental Association*.

[16] Worthington P (2004). Injury to the inferior alveolar nerve during implant placement: a formula for protection of the patient and clinician. *International Journal of Oral and Maxillofacial Implants*.

[17] Xu GZ, Yang C, Fan XD (2013). Anatomic relationship between impacted third mandibular molar and the mandibular canal as the risk factor of inferior alveolar nerve injury. *The British Journal of Oral & Maxillofacial Surgery*.

[18] Seibert JS, Cohen DW (1987). Periodontal considerations in preparation for fixed and removable prosthodontics. *Dental Clinics of North America*.

[19] Wilson DJ (1989). Ridge mapping for determination of alveolar ridge width. *The International Journal of Oral & Maxillofacial Implants*.

[20] AlGhamdi AS, Ciancio SG (2009). Guided tissue regeneration membranes for periodontal regeneration—a literature review. *Journal of the International Academy of Periodontology*.

[21] AlGhamdi AS, Shibly O, Ciancio SG (2010). Osseous grafting—part II: xenografts and alloplasts for periodontal regeneration—a literature review. *Journal of the International Academy of Periodontology*.

[22] Chen LC, Lundgren T, Hallström H, Cherel F (2008). Comparison of different methods of assessing alveolar ridge dimensions prior to dental implant placement. *Journal of Periodontology*.

[23] Sümnig W (1990). Determining the alveolar ridge width in planning treatment with endosseous implants. *Stomatologie der DDR*.

[24] ten Bruggenkate CM, de Rijcke TB, Kraaijenhagen HA, Oosterbeek HS (1994). Ridge mapping. *Implant Dentistry*.

